# Aggressive Central Giant Cell Granuloma: A Case Report

**DOI:** 10.7759/cureus.109573

**Published:** 2026-05-24

**Authors:** R Hariharan, Devotta F Andrew, G Satheesh, S Sakthi, Nithish Kumar

**Affiliations:** 1 Oral and Maxillofacial Surgery, Adhiparasakthi Dental College and Hospital, Melmaruvathur, IND

**Keywords:** benign tumors, central giant cell granuloma, giant cell tumor, jaw lesion, maxillary tumor

## Abstract

Central giant cell granuloma (CGCG) is an uncommon benign intraosseous lesion of the jaws that typically affects the anterior mandible in young females. Aggressive variants may demonstrate rapid growth, cortical expansion, pain, root resorption, and recurrence. This report presents an unusual case of aggressive CGCG involving the posterior maxilla in a 20-year-old female patient who presented with progressive swelling and facial asymmetry. Radiographic evaluation revealed a well-defined expansile lytic lesion involving the right maxilla and maxillary sinus. Differential diagnoses included adenomatoid odontogenic tumor, aneurysmal bone cyst, odontogenic myxoma, ameloblastoma, and Brown tumor of hyperparathyroidism. Histopathological examination confirmed the diagnosis by demonstrating multinucleated giant cells within a fibrocellular stroma with hemorrhagic areas and woven bone formation. The lesion was managed by surgical enucleation, curettage, peripheral osteoplasty, and adjunctive cryotherapy. The postoperative period was uneventful, although mild gingival recession and localized interradicular bone loss were observed at two-month follow-up.

## Introduction

Jaffe first described central giant cell granuloma (CGCG) in 1953 [1]. It consists of cellular fibrous tissue with hemorrhagic foci, multinucleated giant cells, and occasional trabeculae of woven bone. Although benign, it can be locally aggressive and destructive. CGCG most commonly affects the mandible, followed by the maxilla, with female predominance in individuals younger than 30 years of age. CGCG is a relatively uncommon intraosseous lesion accounting for less than 7% of all benign jaw tumors. Its etiology remains controversial, with proposed causes including reactive, inflammatory, infective, or neoplastic processes, often following trauma [2]. Aggressive and non-aggressive lesions can be differentiated based on clinical signs, symptoms, and histological features. Aggressive lesions are characterized by rapid growth, cortical expansion, cortical perforation, root resorption, tooth displacement, facial asymmetry, pain, and a higher tendency for recurrence following treatment. Radiographically, CGCG may mimic several odontogenic and non-odontogenic lesions, making histopathological evaluation essential for definitive diagnosis. Posterior maxillary involvement is relatively uncommon and poses additional diagnostic and surgical challenges due to its proximity to the maxillary sinus and orbital floor.

In this case report, we highlight the diagnostic challenges associated with aggressive CGCG in an uncommon posterior maxillary location and emphasize the importance of histopathological confirmation, a comprehensive differential diagnosis, and treatment modalities, as well as post-treatment complications and outcomes.

## Case presentation

**Figure 1:**
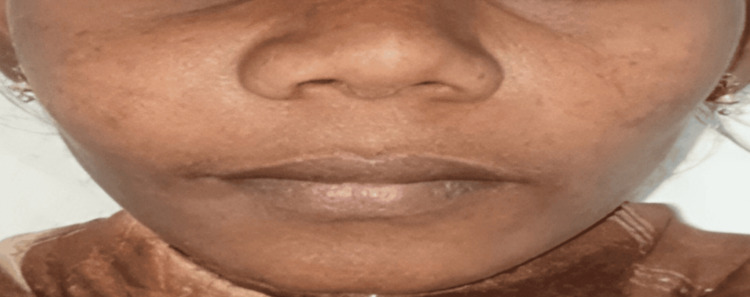
Extraoral image showing gross facial asymmetry over the right malar area with obliteration of the nasolabial fold

A 20-year-old female patient presented to the Department of Oral and Maxillofacial Surgery with a six-month history of pain and swelling in the right cheek region. Extraoral examination revealed gross facial asymmetry due to a swelling over the right malar area, with obliteration of the nasolabial fold (Figure 1).

**Figure 2:**
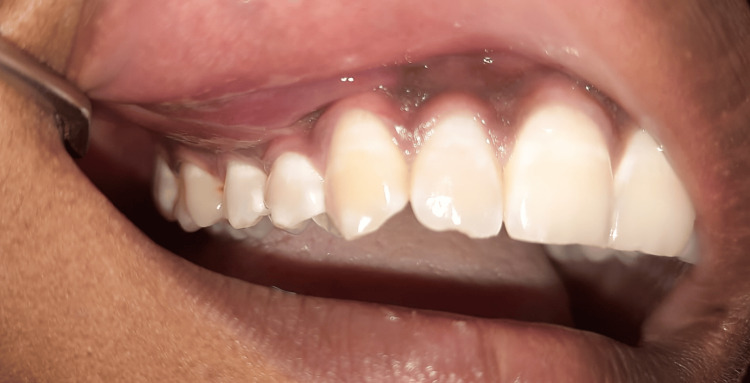
Intraoral image showing obliteration of buccal vestibule of posterior region

Intraorally, there was obliteration of the buccal vestibule in the region of teeth 14, 15, and 16, without tenderness (Figure 2). Pulp vitality testing was assessed using gutta-percha sticks, which revealed that the adjacent teeth were vital. Based on the clinical and radiographic findings, differential diagnoses included adenomatoid odontogenic tumor, odontogenic keratocyst, calcifying epithelial odontogenic cyst, nasolabial cyst, radicular cyst, giant cell tumor of bone, and CGCG.

**Figure 3:**
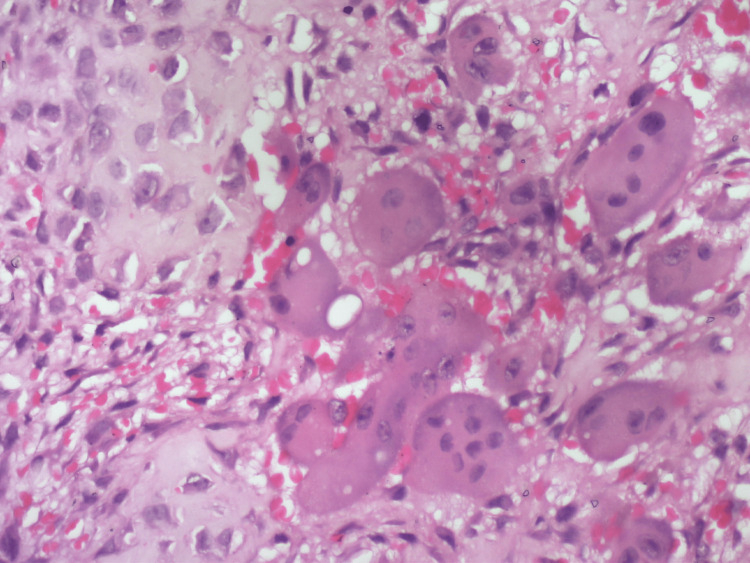
H&E-stained section showing giant cells (40x) predominantly clustered (diffuse pattern) around hemorrhagic areas and contain approximately 5-20 nuclei per cells H&E: hematoxylin and eosin

Under local anesthesia, an incisional biopsy was performed by creating a bony window into the right maxillary sinus, through which a mixed specimen of hard and soft tissue was obtained for histopathological evaluation. Histopathological analysis of the biopsy specimen revealed highly cellular fibrovascular stroma containing numerous irregularly distributed multinucleated osteoclast-like giant cells, often clustered around hemorrhagic areas. The background consists of spindle-shaped fibroblastic mesenchymal cells with areas of hemorrhage, extravasated RBCs, hemosiderin deposition, and occasional reactive woven bone trabeculae. No significant cellular atypia, pleomorphism, atypical mitoses, or necrosis is identified, thereby confirming the diagnosis of CGCG (Figure 3).

**Figure 4:**
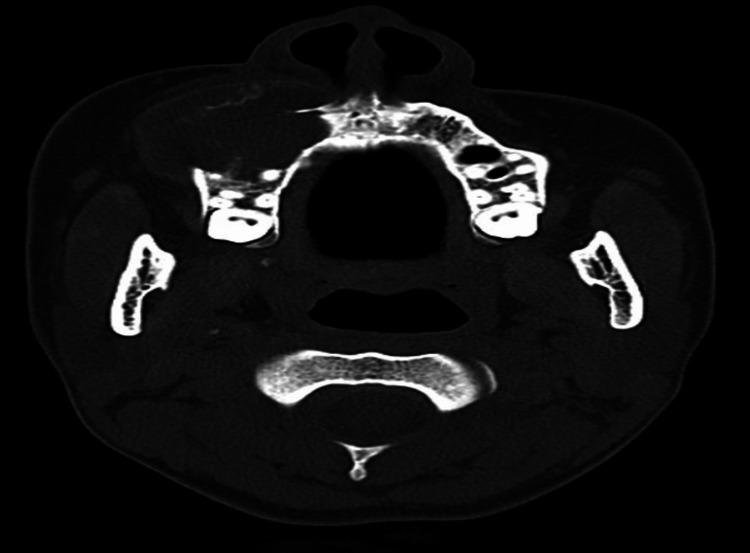
Axial section CT demonstrating a well-defined mixed radiolucent-radiopaque expansile lesion of size 3.5 × 3.4 × 3.8 cm (AP × ML × SI), destruction of labial, buccal cortical plate, and thinning of palatal cortical plate CT: computed tomography, AP: anteroposterior, ML: mediolateral, SI: superoinferior

CT of the facial bones revealed an expansile osteolytic lesion involving the right maxilla with complete opacification and associated soft tissue density within the right maxillary sinus measuring approximately 3.5 × 3.4 × 3.8 cm (AP × ML × SI), with multiple thin bony septations. The lesion was centered in the right maxilla and extended superiorly into the right maxillary sinus, abutting the floor of the right orbit without evidence of orbital floor erosion. Medially, the lesion abutted the uncinate process and the medial wall of the right orbit, resulting in obstruction of the right osteomeatal unit. Inferiorly, there was cortical destruction and thinning of the sinus walls, with expansion into the adjacent alveolar process with involvement around posterior maxillary teeth, and likely erosion of the medial and anterior walls of the maxillary sinus. Presence of internal septae/multiloculated appearance in parts of the lesion. Anteroposteriorly, the mass extended up to the buccal mucosa, although definitive involvement of the mucosa could not be established. The lesion appears more aggressive than a simple inflammatory cyst because of bony destruction, expansile growth, and loss of normal sinus architecture (Figure 4).

**Figure 5:**
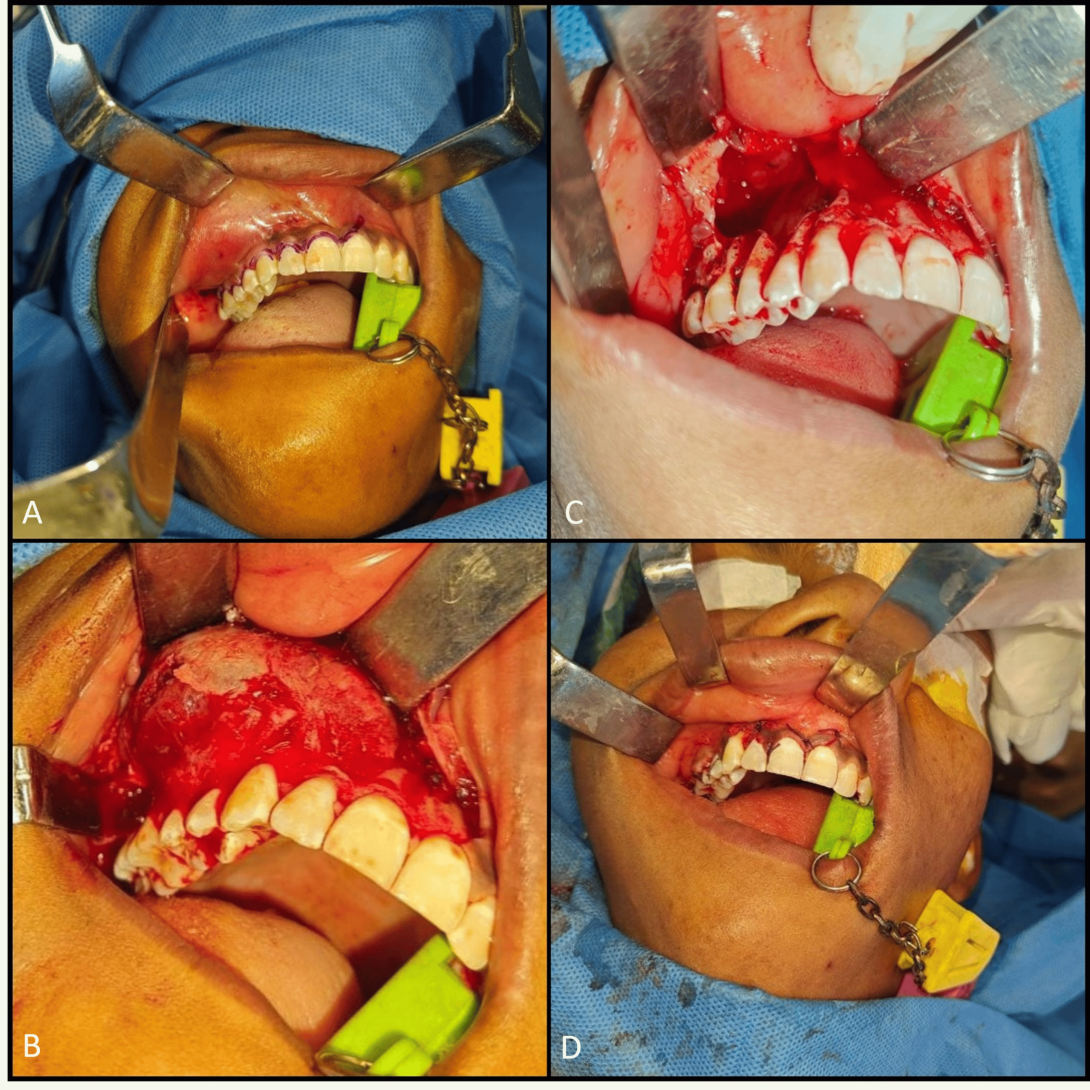
Intraoperative images (A) Intraoral preoperative view showing vestibular incision marking over the anterior maxillary lesion. (B) Intraoperative exposure demonstrating the expansile bony lesion after flap reflection. (C) Surgical cavity following curettage and enucleation of the lesion. (D) Immediate postoperative view after closure of the surgical site.

**Figure 6:**
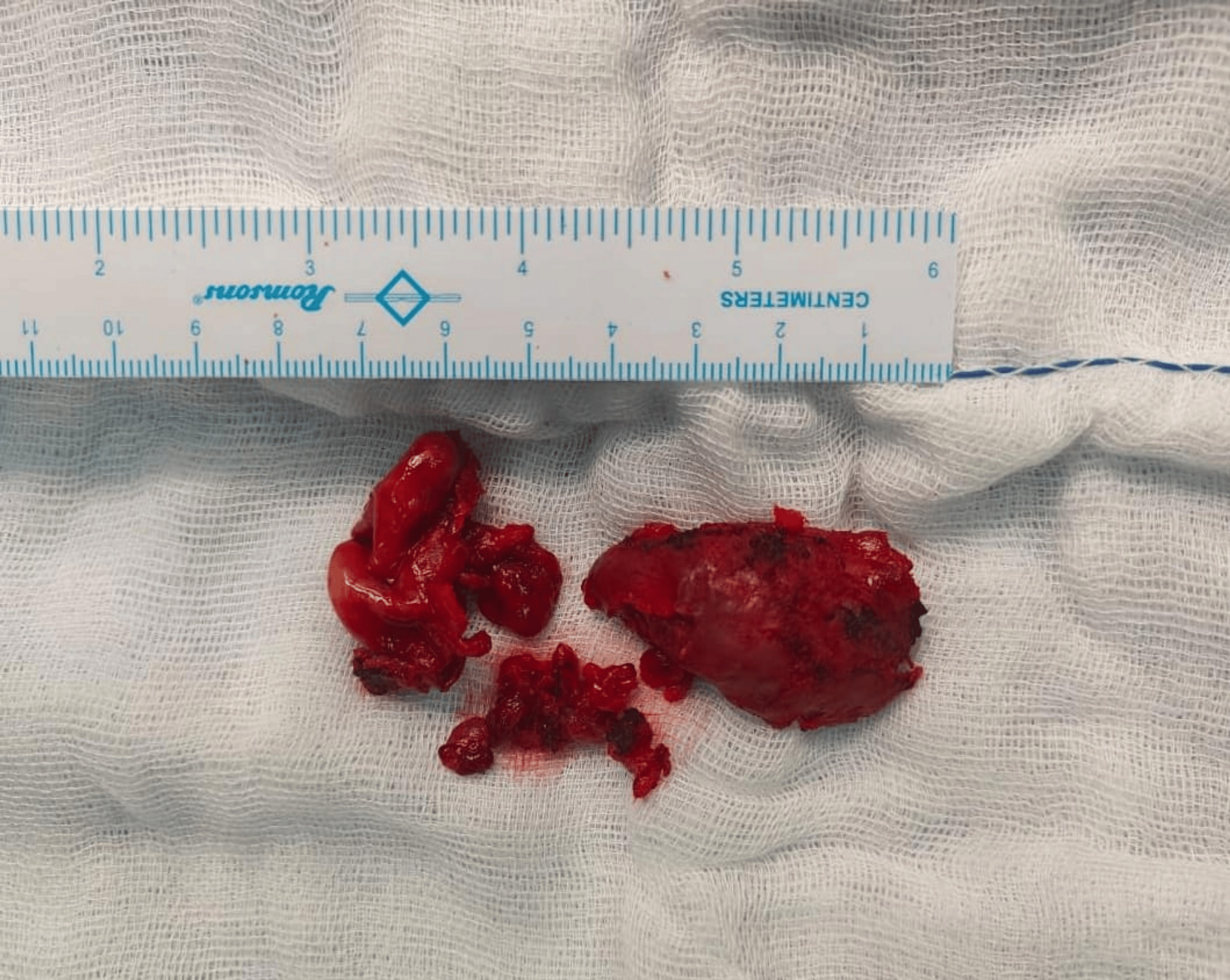
Excised lesion

Routine preoperative investigations, including thyroid function tests, serum calcium, phosphorus, and alkaline phosphatase levels, were conducted to rule out Brown’s tumor, a differential diagnosis associated with hyperparathyroidism. All biochemical parameters were within normal limits, effectively excluding Brown’s tumor (Table 1). Surgical enucleation of the lesion was planned under general anesthesia. Local infiltration with 2% lignocaine containing 1:200,000 adrenaline was administered to achieve hemostasis and analgesia. A crevicular incision was made, and a mucoperiosteal flap was elevated to expose the lesion. The tumor mass was enucleated in toto, followed by thorough curettage of the surrounding granulation tissue (Figures 5-6).

**Table 1:** Biochemical parameters within limits Normal serum calcium, phosphorus, alkaline phosphatase, and thyroid function test values helped exclude Brown's tumor associated with hyperparathyroidism.

Investigation	Patient value	Normal range
Serum calcium (total)	9.22 mg/dl	8.5-10.5 mg/dl
Serum phosphorus	4.28 mg/dl	2.5-4.5 mg/dl
Serum alkaline phosphatase	85.90 IU/L	44-147 IU/L
Total T3 (TT3)	1.24 ng/mL	0.7-2.04 ng/mL
Total T4 (TT4)	8.45	4.6-10.5 mg/dL

To minimize the risk of recurrence, cryotherapy was carried out following surgical curettage of the lesion (Table 2). After achieving adequate exposure and hemostasis, the cryoprobe was applied in direct contact with the bony cavity walls, and residual lesion-site cryotherapy was performed using a liquid-nitrogen cryoprobe (Ascon Medical Instruments PVT. LTD., India) capable of achieving temperatures up to −196°C. The lesion site underwent repeated freeze-thaw cycles using the contact probe technique, with protection of adjacent vital structures. Postoperatively, satisfactory healing was observed without immediate complications, and the prognosis was considered favorable, with periodic follow-up to evaluate bone healing and recurrence.

**Table 2:** Steps in cryotherapy performed

	Steps in cryotherapy (cryoprobe)
1.	Application of the cryoprobe: The cryoprobe tip was placed directly over the lesion with gentle pressure to ensure complete contact with the tissue surface.
2.	Initiation of freezing: Cryogen flow was activated, rapidly cooling the probe tip and surrounding tissue.
3.	Formation of ice ball: Freezing was continued until a visible ice ball formed, extending slightly beyond the lesion margins to ensure adequate tissue destruction.
4.	Maintenance of freeze cycle: The lesion was maintained in the frozen state for approximately 20-60 seconds, depending on lesion size and depth.
5.	Thawing phase: Cryogen flow was stopped, allowing the tissue to thaw spontaneously at room temperature.
6.	Repeat freeze-thaw cycle: A second and/or third freeze–thaw cycle was performed to enhance cellular necrosis and improve treatment efficacy.
7.	Assessment of tissue response: Adequate freezing was confirmed by tissue blanching and formation of a uniform frozen zone.
8.	Removal of cryoprobe: After complete thawing, the cryoprobe was gently detached from the tissue to avoid mechanical injury.

**Figure 7:**
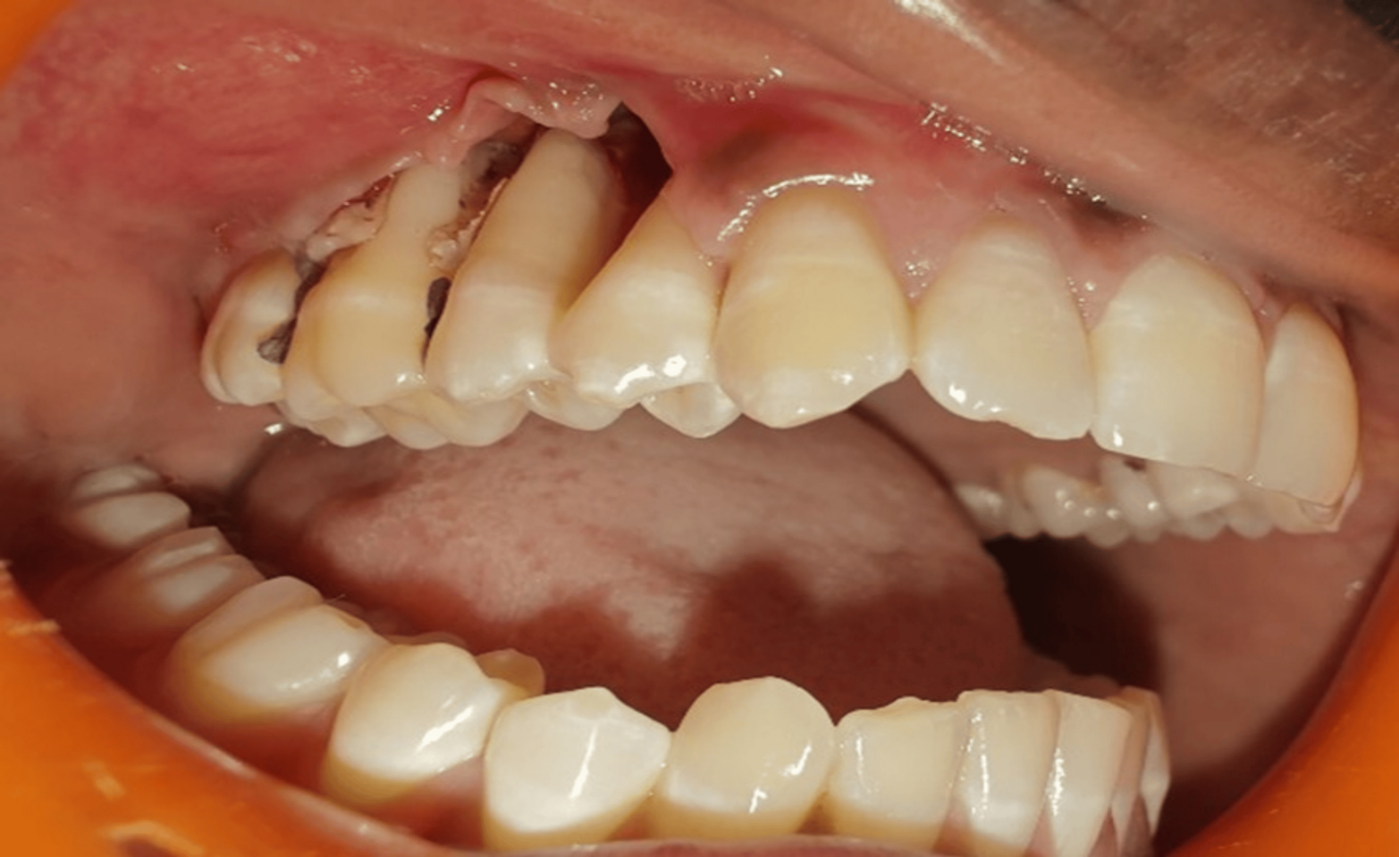
Postoperative image

The surgical site was then irrigated thoroughly with 0.9% sodium chloride (normal saline), and peripheral osteoplasty was performed to smooth sharp bony margins and recontour the area. Wound closure was completed, and a zinc oxide eugenol pack (GC Dental, India) was placed and removed after seven days. The patient was advised to perform steam inhalation and was prescribed antihistamines (tab. levocetirizine 5 mg for seven days) for symptomatic relief. At the two-month postoperative follow-up, the patient exhibited gingival recession about teeth 15 and 16, with loss of bony support in the region. No additional surgical procedure was performed for the associated bone loss and gingival recession in the affected region at the time of primary surgery. The patient has been kept under periodic follow-up to assess postoperative healing and residual periodontal and soft tissue defects. Future periodontal or reconstructive management will be determined by clinical healing and esthetic/functional requirements. However, there was no evidence of oro-nasal communication (Figure 7).

## Discussion

CGCG is a benign intraosseous lesion of the jaws characterized histologically by multinucleated giant cells in a background of spindle-shaped stromal cells. Despite its non-neoplastic nature, CGCG can exhibit locally aggressive behavior, particularly in younger individuals, which poses a diagnostic and therapeutic challenge. The lesion is more prevalent in females under 30 years of age and predominantly affects the anterior mandible; however, maxillary involvement, especially posteriorly, as in our case, is also documented [1,2].

Chrcanovic et al. performed a systematic review of central giant cell lesions of the jaws involving 365 studies and 2,270 lesions. The overall recurrence rate was 17.6%, with aggressive lesions showing higher recurrence (22.8%) than non-aggressive lesions (7.8%) after surgery. Pharmacological therapy achieved partial or complete regression in most cases, though aggressive lesions responded less favorably to corticosteroids. Conservative procedures such as curettage, enucleation, and marginal resection showed higher recurrence, especially in lesions with aggressive behavior, cortical perforation, and root resorption. Evaluation of combined surgical and pharmacological therapy remained limited due to heterogeneous treatment protocols [2].

The etiology of CGCG remains uncertain; however, several hypotheses suggest a reactive, inflammatory, or vascular origin. Molecular studies have identified overexpression of RANKL and MMP-9, suggesting osteoclast-like activity in lesion progression [3,4]. Additionally, recent genetic investigations have highlighted somatic mutations in TRPV4, KRAS, and FGFR1 genes in a subset of aggressive CGCGs, indicating a neoplastic component in some cases [5]. Radiographically, CGCG can present as unilocular or multilocular radiolucencies with cortical expansion and root resorption, making it radiologically similar to other giant cell lesions, such as aneurysmal bone cysts or brown tumors of hyperparathyroidism. Histopathology remains the gold standard for diagnosis. In our case, histological examination revealed the classical features of CGCG with no signs of cellular atypia, supporting a non-neoplastic diagnosis.

Therapeutic approaches vary based on lesion size, location, and aggressiveness. Curettage remains the mainstay of treatment for non-aggressive forms, although recurrence rates of 11-49% have been reported, particularly in lesions with aggressive radiographic features [6]. Alternative therapies such as intralesional corticosteroid injections, calcitonin, interferon-α, and, more recently, denosumab have been proposed, especially for lesions in surgically challenging sites or in younger patients to avoid mutilating surgery [7,8]. Denosumab, a RANKL inhibitor, has shown promising results in aggressive CGCG, with reduced lesion volume and recurrence. However, long-term follow-up is essential due to potential side effects, including osteonecrosis of the jaw [9]. In recent years, a molecular classification of CGCG and related giant cell lesions has been proposed, which may aid in distinguishing between reactive and neoplastic subtypes and guiding personalized treatment strategies [10]. Immunohistochemical markers such as p63, Ki-67, and RANKL are being explored to assess the proliferative and osteoclastic activity within the lesion [11].

Cryotherapy has been used as a conservative adjunct following curettage or peripheral ostectomy for aggressive jaw lesions to reduce recurrence. Cryotherapy induces cellular necrosis and vascular thrombosis, thereby destroying residual pathological tissue while preserving the inorganic bony framework, which facilitates postoperative bone regeneration and healing [12]. In this case, we have utilized cryotherapy as an adjuvant modality of treatment alongside enucleation and curettage to prevent recurrence.

## Conclusions

The lesion in the present case was classified as aggressive CGCG based on established clinicoradiographic criteria, including painful swelling, rapid progression over six months, cortical expansion, involvement of the maxillary sinus, and significant lesion size. Although root resorption was absent, the expansile behavior and anatomical extension supported aggressive biological behavior. Based on the previous study, we have performed surgical enucleation and peripheral osteotomy. Additionally, cryotherapy has been performed under aseptic conditions. Postoperatively, our patient has no functional impairment. In our case, the patient was followed up for three months, which is relatively short, and was subsequently lost to follow-up. Future directions should focus on integrating molecular diagnostics with histopathological evaluation to refine the classification, prognosis, and treatment of CGCG. Early diagnosis and comprehensive management are essential to minimize complications, prevent recurrence, and preserve surrounding anatomical structures. Regular postoperative follow-up is crucial for monitoring healing and detecting any early signs of recurrence or functional impairment.
